# Targeting the Ubiquitin–Proteasome System for Cancer

**DOI:** 10.1002/mco2.70391

**Published:** 2025-10-03

**Authors:** Zhaoyun Liu, Jiao Lai, Ziyu Ma, Jianhua Pan, Chun Yang, Rong Fu

**Affiliations:** ^1^ Department of Hematology Tianjin Medical University General Hospital Tianjin Key Laboratory of Bone Marrow Failure and Malignant Hemopoietic Clone Control Tianjin Institute of Hematology State Key Laboratory of Experimental Hematology Tianjin China

**Keywords:** ubiquitin, ubiquitin–protease, ubiquitination

## Abstract

Ubiquitin is a highly conserved small molecule that exists in large quantities in eukaryotic cells and plays a crucial role in protein quality control by phagocytosis and degradation of ubiquitin‐modified proteins. The abnormal expression of the ubiquitin–proteasome system (UPS) in cancer leads to the abnormal expression of ubiquitin ligases and ubiquitin‐binding enzymes, resulting in the abnormal accumulation of ubiquitinated proteins. Consequently, UPS dysregulation can contribute to tumor initiation, progression, and resistance to therapy. While proteasome inhibitors have shown clinical success, comprehensive reviews integrating upstream UPS components and their therapeutic potential are lacking. This paper reviews the composition of the UPS, its tumor‐promoting mechanisms, as well as the small molecule inhibitors and proteasome inhibitors based on this system, including their mechanisms of action and adverse effects, and explores their clinical advances in the treatment of cancer. This review provides a valuable framework for developing next‐generation anti‐cancer therapies and establishes the UPS as a critical therapeutic target for precision oncology.

## Graphical abstract

This figure shows the mechanism of protein degradation by ubiquitin–proteasome system. The process begins with ubiquitin activation by the E1 enzyme, forming a thioester bond between E1's cysteine residue and ubiquitin's glycine carboxyl group, powered by ATP hydrolysis. The activated ubiquitin is then transferred to E2 via trans‐esterification, binding to E2's active‐site cysteine. Finally, E3 ligase facilitates the transfer of ubiquitin from E2 to a lysine residue on the target protein, forming an isopeptide bond and releasing E2, resulting in a ubiquitinated substrate ready for proteasomal degradation.

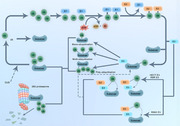

## 1 Introduction

The history of the ubiquitin–proteasome system (UPS) dates back to the 1980s, when scientists discovered a new form of protein modification—ubiquitination, which is a process by which ubiquitin molecules label a target protein, leading to its degradation [1]. With progressive research, the role of the UPS in cell cycle, transcription, oxidative stress, and autophagy was gradually recognized. Since the 1990s, the association of UPS with various diseases has been revealed, especially in the field of cancer, where ubiquitination has been shown to be closely related to tumorigenesis and progression [2].

The UPS is a complex, multi‐step mechanism involving various proteins [3]. Initially, proteins targeted for degradation are marked by ubiquitin, a small regulatory polypeptide. This tagged protein is subsequently identified and broken down by the proteasome [4]. The UPS ensures that cells eliminate unnecessary or damaged proteins with notable precision. Essential elements of the system encompass ubiquitin, the ubiquitin‐activating enzyme E1, ubiquitin‐conjugating enzymes E2, ubiquitin ligases E3, the 26S proteasome, and deubiquitinating enzymes [5].

Current studies suggest that the UPS profoundly influences cancer progression via multiple mechanisms, and the widespread proteasome modification in cancer tissues reveals a strong link between ubiquitination and diverse biological functions in cancer [6, 7]. Ubiquitination has emerged as a target for cancer therapy, particularly for multiple myeloma (MM), where it has been demonstrated that UPS inhibitors (e.g., bortezomib (BTZ), carfilzomib, and ixazomib) are first‐line therapeutic agents [8, 9]. In recent years, targeted protein degradation has emerged as an innovative chemical probe and drug discovery approach using small molecules or biological agents to direct proteins toward cellular degradation mechanisms, with cereblon and VHL playing a central role in UPS‐dependent targeted protein degradation as E3 ligases. In particular, proteolysis‐targeting chimaera (PROTAC) technology has opened new avenues for cancer therapy by selectively degrading key oncogenic proteins via the UPS [10]. Moreover, the expression levels of UPS‐related genes are also closely related to the prognosis of patients with certain types of cancer, further highlighting their importance in cancer biology [11]. In summary, the UPS occupies a central position in cancer research and therapy, and its research scope not only covers the basic biological mechanisms but also extensively involves drug development and clinical applications [12]. With continuous research, the UPS is expected to provide more innovative strategies and new drugs for cancer treatment [13].

The UPS has demonstrated its complex and multidimensional role in cancer research [14]. This review will discuss the structure of the UPS and its mechanism of action in tumors, some of the cutting‐edge therapeutic strategies developed against its tumors, and particularly the targeting of MM.

## 2 Mechanisms of the UPS

### 2.1 Ubiquitination

Ubiquitin, a diminutive protein made up of 76 amino acids, is exclusively present in eukaryotic cells [15]. This spherical, highly conserved, and heat‐resistant protein primarily plays a role in the degradation of cellular proteins [16]. Ubiquitin serves as a “tag” inside the cell, labeling proteins that need to be degraded and directing these proteins to the “garbage disposal station” inside the cell, which is the proteasome, for degradation [17].

**FIGURE 1:**
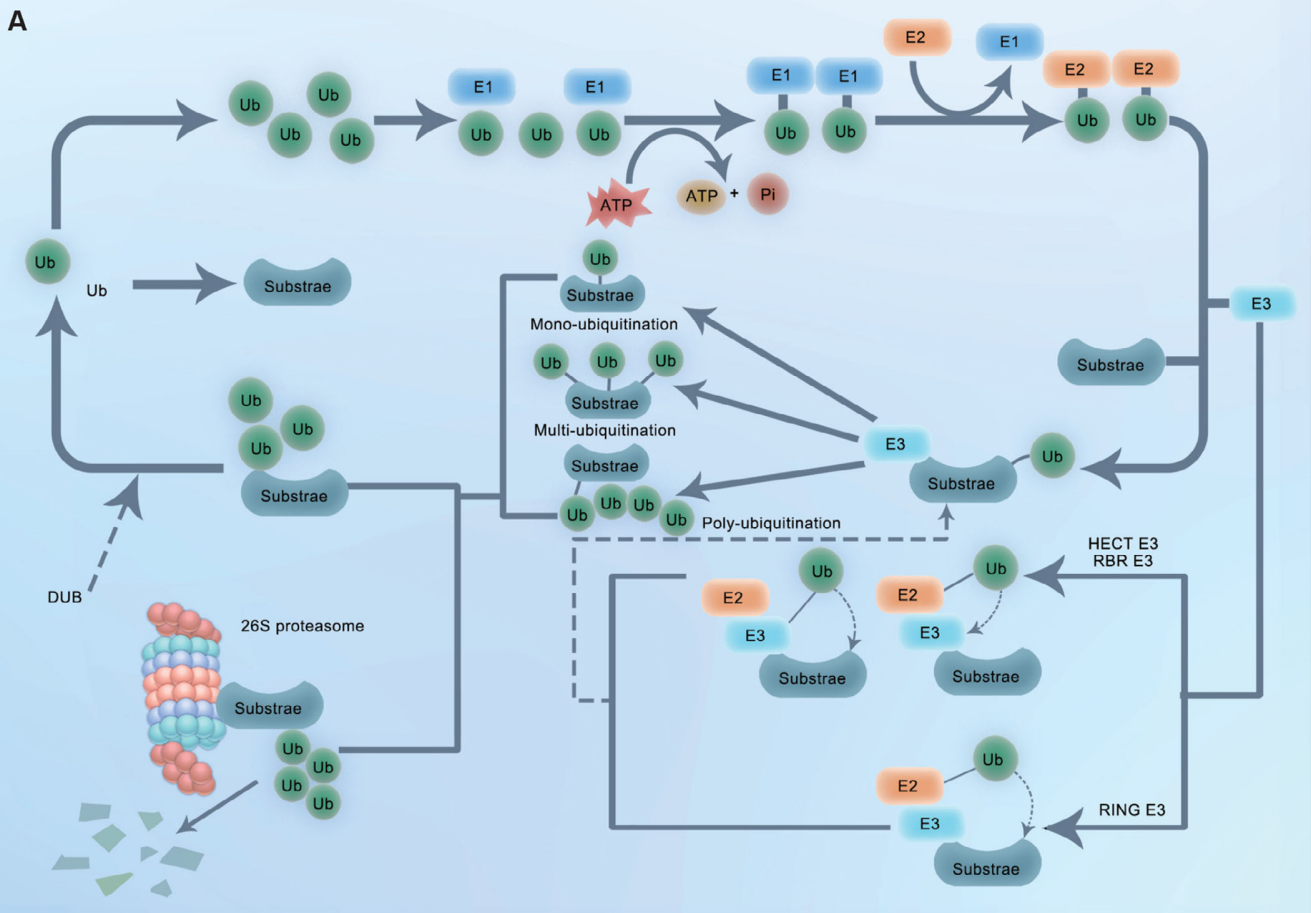
This figure shows the mechanism of protein degradation by ubiquitin–proteasome system. The process begins with ubiquitin activation by E1 enzyme, forming a thioester bond between E1's cysteine residue and ubiquitin's glycine carboxyl group, powered by ATP hydrolysis. The activated ubiquitin is then transferred to E2 via trans‐esterification, binding to E2's active‐site cysteine. Finally, E3 ligase facilitates the transfer of ubiquitin from E2 to a lysine residue on the target protein, forming an isopeptide bond and releasing E2, resulting in a ubiquitinated substrate ready for proteasomal degradation.

Ubiquitination encompasses a sequence of processes that involve ubiquitin molecules, in addition to enzymes that activate ubiquitin (E1) and conjugate ubiquitin (E2), as well as ubiquitin ligases (E3) [18]. Ubiquitination is a highly dynamic post‐translational modification of proteins commonly occurring in organisms (Figure 1) [19]. The ubiquitin molecule is activated, and the thiol group of E1 connects to the glycine carboxyl group of the ubiquitin molecule. Adenosine triphosphate (ATP) provides energy to this process, ultimately forming a thioester bond between ubiquitin and E1. E1 then hands over the activated ubiquitin to E2 via esterification, and ubiquitin binds to the cysteine residue at the active site of E2. E2 plays a role in ubiquitin transfer from E1 to E3 [20]. Finally, E3 connects the ubiquitin bound to E2 to the target protein and releases E2, forming a specific ubiquitinated protein [21, 22].

Ubiquitination plays an indispensable role in vital processes such as cell apoptosis, differentiation, the life cycle, and immune responses [23]. Many proteins have a short life cycle within cells and need to be rapidly degraded. Ubiquitination is a method of labeling proteins so that they can be quickly recognized and degraded by proteasomes [24]. This regulatory mechanism effectively and rapidly eliminates unwanted or incorrectly expressed proteins and maintains intracellular homeostasis [25, 26]. Furthermore, to sustain normal cell growth and division, some proteins must be destroyed at specific moments during the cell cycle. Ubiquitination plays a crucial role in activating cell cycle checkpoints and degrading cyclins [27]. In addition, infected cells label viral or bacterial proteins via ubiquitination [28], which guides the immune system in recognizing and clearing these infections [29, 30]. Ubiquitination is also involved in immune response regulation; for example, in the T‐cell immune response, the signaling of T‐cell receptors requires ubiquitination modification [31]. Ubiquitination also participates in signal transduction processes, in which key signaling molecules must be rapidly degraded to terminate signal transduction [32]. Ubiquitination can label these signaling molecules, allowing them to be rapidly degraded after signal transduction is complete [33]. Finally, some proteins require degradation at specific time points during cell differentiation to maintain specific cellular functions.

### 2.2 UPS

In eukaryotic cells, the UPS functions as an ATP‐reliant and distinctly selective process for breaking down proteins [34]. It plays a key role in removing aged, damaged, or misfolded proteins and is involved in the regulation of several critical biological processes, including inflammation, immune responses, cell cycle control, and signal transduction [35]. The UPS is made up of several essential components. (1) Ubiquitin molecule: Activated ubiquitin attaches to substrate proteins via a series of enzymatic reactions, forming a ubiquitin chain that signals the protein for degradation by proteasomes [36]. (2) Ubiquitin‐activating enzyme (E1): Using ATP, E1 facilitates the formation of a thioester bond between a cysteine residue in its active site and the glycine at the C‐terminus of ubiquitin, thereby activating ubiquitin. (3) Ubiquitin‐conjugating enzyme (E2): E2 interacts with activated ubiquitin through its cysteine active site and mediates the transfer of ubiquitin to proteins marked for degradation. (4) Ubiquitin ligase (E3): E3 enzymes play a critical role by specifically identifying target proteins and catalyzing their polyubiquitination, making E3 a pivotal player in the UPS [37]. (5) Proteasomes: These cylindrical structures, which are located in the cytoplasm and nucleus of eukaryotic cells, play a crucial role in the breakdown of ubiquitinated proteins via an ATP‐dependent process. The 26S proteasome consists of a 20S proteolytic core, flanked by two 19S regulatory units that regulate substrate recognition and processing. This system is essential for preserving protein balance in cells, and its malfunction is associated with various illnesses, including cancer [38].

The UPS can degrade many short‐lived or misfolded proteins, which is important for maintaining protein balance and normal cellular function [39]. The UPS in cells recognizes target proteins that need to be degraded. Typically, ubiquitin molecules alter these target proteins, making them more recognizable by the proteasomal system and allowing their breakdown. The 20S core and 19S regulatory particles comprising the 26S proteasome complex are assembled in the cytoplasm [40], and 19S regulates the binding of particles to the target proteins to be degraded, promoting their entry into the 20S core particles (CPs) [29]. The target protein is broken down into short peptide fragments as soon as it enters the 20S CPs. After additional breakdown into amino acids, these peptide fragments are expelled from the cytoplasm [41]. After the degradation products are exported to the cytoplasm, amino acids can be reused to synthesize novel proteins, whereas small peptide segments can be further degraded or excreted outside the cell. In summary, the normal operation of the UPS involves the degradation of damaged, mutated, or excess proteins within cells to maintain intracellular protein homeostasis and help cells resist various stress stimuli [42].

MM is a malignant disorder arising from plasma cells in the bone marrow. The development and progression of MM are closely associated with the regulation of the UPS [43]. Several proteins involved in cell proliferation and apoptosis, which are crucial in MM, are regulated by the UPS, as their phagocytosis and degradation are tightly controlled by this system. Excessive proteasomal activity is a significant factor contributing to MM pathogenesis [44]. Consequently, the UPS plays a critical role in the onset, progression, and treatment of MM.

The treatment of MM has always been a prominent subject in contemporary medical research. Investigation of the UPS in MM treatment has drawn increasing attention in recent years. Tumor cell survival, growth, and medication resistance are strongly correlated with aberrant activation of the UPS. Consequently, blocking the activity of the UPS may alter the biological behavior of tumor cells [45]. This provides new insights and methods in the treatment of MM. Currently, there exist drugs targeting the ubiquitin–targeted chimeras，and molecular gels. These medicines can exert anti‐tumor effects through the induction of tumor cell death and the inhibition of cell cycle progression [46]. Multiple clinical trials have demonstrated the good therapeutic and safety effects on MM [47, 48].

However, the UPS system has adverse effects on the treatment of MM. Several drugs targeting the UPS are currently entering clinical trial stages. Among them, the most representative drugs are inhibitors targeting ubiquitin ligase E3, such as thalidomide and its analogues, the common adverse reactions of these drugs are gastrointestinal reactions, bone marrow suppression, and immunosuppression. Additionally, because of the important physiological function of the UPS in cells, inhibitors targeting this system may have toxic effects on normal cells [49]. Therefore, strict control of drug dosage and administration methods is necessary in clinical applications to reduce the incidence of adverse reactions. This article introduces various treatment methods and research progress for cancer based on the ubiquitination and proteasome systems, including their mechanisms of action, clinical trials, and adverse reactions, and explores future development directions.

## 3 Mechanisms of the UPS in Cancer

### 3.1 Excessive Degradation of Tumor Suppressor Proteins

The UPS can affect the survival of tumor cells by promoting the degradation of oncoproteins. For example, the oncoprotein p53 is the “gene guardian” of the cell and is essential for maintaining genome stability. Through ubiquitin ligases and deubiquitinating enzymes, the UPS can regulate the degradation rate of p53 [50]. When the UPS functions abnormally, the degradation of p53 is blocked, leading to its accumulation in cells, which in turn triggers anti‐tumor responses such as cell cycle arrest and apoptosis. The ubiquitination and degradation of p53 are primarily controlled by MDM2, an E3 ubiquitin ligase, and stress signals lead to the stabilization of p53 by altering the post‐translational modifications or protein–protein interactions of MDM2 and p53 [51]. However, in tumor cells, the UPS tends to promote tumorigenesis and progression by facilitating p53 degradation and allowing tumor cells to escape apoptosis. For example, frequent mutations of p53 in osteosarcoma lead to its aberrant degradation by the UPS, accelerating tumor progression and metastasis [52]. In addition, UBE2T, as an E2 ubiquitin‐conjugating enzyme, is involved in the malignant transformation of various cancers, such as lung and gastric cancers, by facilitating ubiquitylation modification of tumor suppressors [53].

### 3.2 Regulation of Oncogenic Protein Degradation

The UPS can also promote tumorigenesis by blocking the degradation of oncogenic proteins [54]. Oncogenic proteins are often overexpressed in tumor cells and play roles such as promoting cell proliferation and inhibiting apoptosis. Through its ubiquitin ligases, the UPS recognizes and binds these oncogenic proteins and promotes their degradation. However, in tumor cells, the function of ubiquitin ligases may be impaired, leading to the accumulation of oncogenic proteins within the cell, which in turn promotes tumor development. RB family proteins are important tumor suppressors, and their stability is regulated by the UPS, the dysregulation of which is closely related to pathogenesis [55, 56].

### 3.3 Cell Cycle Control and DNA Damage Repair

The major regulators of the cell cycle include cyclin‐dependent kinase (CDK) and cyclin. The UPS precisely regulates the cell cycle by degrading key cell cycle regulators such as cyclin and CDK inhibitory proteins [57]. For example, during the transition from G1 to S phase, cyclin D binds to CDK4/6 to form a complex that facilitates cell entry into the S phase. When cyclin D completes its mission, the UPS degrades it, ensuring an orderly cell cycle [58]. UPS dysfunction can accelerate the degradation of oncogenic proteins such as p53, which is an important tumor suppressor protein that prevents malignant cell proliferation by inducing cell cycle arrest or apoptosis in response to stress signals such as DNA damage [59]. When p53 is abnormally degraded, its cancer‐suppressing function is lost, increasing the risk of cells becoming cancerous [60]. UPS dysfunction may also lead to the accumulation of oncogenic proteins (e.g. c‐Myc and c‐Jun) in cells [61]. These proteins usually promote cell proliferation and inhibit apoptosis, among other functions. Their abnormal accumulation disturbs the normal regulation of the cell cycle and promotes malignant cell proliferation. UPS dysfunction may also affect the degradation of other key regulators of the cell cycle, such as cyclin E and CDK2. Abnormal expression or impaired degradation of these factors can disturb the normal course of the cell cycle and promote tumorigenesis. The UPS exerts important effects on cell growth, proliferation, and apoptosis by precisely regulating the degradation of key regulatory factors in the cell cycle [62]. Dysfunction of this system can lead to the accelerated degradation of oncogenic proteins, accumulation of oncogenic proteins, and abnormalities in cell cycle regulators, thus promoting tumor development and progression [63].

### 3.4 Regulation of Apoptosis and Autophagy

The UPS affects tumor cell survival by regulating the degradation of apoptosis and autophagy‐related proteins [64]. For example, Bcl‐2 family proteins are key proteins in the regulation of apoptosis. The UPS inhibits apoptosis by promoting the degradation of pro‐apoptotic proteins [65]. The UPS can also affect the level of autophagy in tumor cells by regulating the degradation of autophagy‐related proteins [66]. In tumor cells, the UPS tends to promote tumor development and progression by inhibiting apoptosis and promoting autophagy, causing tumor cells to evade apoptosis [67].

### 3.5 Regulation of Signal Transduction Pathways

The UPS affects tumor cell growth and metastasis by regulating key proteins in signal transduction pathways. For example, signal transducer and activator of transcription (STAT) plays an important role in the growth, proliferation, and metastasis of tumor cells. The UPS inhibits tumorigenesis and progression by promoting the degradation of STAT proteins and inhibiting the activation of their downstream signaling pathways [68]. However, in tumor cells, these regulatory mechanisms may fail, leading to over‐activation of the STAT signaling pathway and the promotion of tumor growth and metastasis [69].

After investigating the mechanism of action of the UPS in tumors, we found that this system is critical for the growth, proliferation, and maintenance of protein homeostasis of tumor cells. Given its critical position, targeting the UPS has emerged as a new strategy for tumor therapy. By specifically interfering with UPS function, the survival mechanism of tumor cells can be effectively perturbed. Next, this article will elaborate on the therapeutic strategies and recent advances in targeting the UPS for tumors.

## 4 Targeting the UPS as a Strategy for Cancer Treatment

### 4.1 Treatment of Cancer Based on the Ubiquitination System

The components of the ubiquitination system include ubiquitin, ubiquitin ligase E3, ubiquitin‐conjugating enzyme E2, and ubiquitin‐activating enzyme E1. Dysfunction of the ubiquitination system is closely related to the condition of the cancer patient. Abnormalities are present in the ubiquitination system of tumor cells, including abnormal expression of ubiquitin‐binding enzymes and ligases, as well as the accumulation of ubiquitinated proteins (the ubiquitination system in tumor cells exhibits abnormalities, characterized by the deregulated expression of ubiquitin‐binding enzymes and ligases, along with the accumulation of ubiquitinated proteins) [47]. The UPS is crucial for oncogenesis, progression, and treatment. A key approach in cancer treatment involves targeting the ubiquitination system (Table 1). The ubiquitination of tumor cells can be influenced by regulating the expression of E1, E2, and E3, and the ubiquitination process of tumor cells can be influenced, thereby inhibiting tumor cell proliferation and promoting apoptosis [70], Table 2.

**TABLE 1:** Classification, mechanisms, targets, and therapeutic applications of ubiquitination system inhibitors.

Drug	Synonym	Target	Mechanisms	Cancer types	Treatments	Phase Status	Clinical trial numbers
MLN7243	TAK‐243	E1 UBA1	Blocks the binding of the ubiquitin molecules to the E1 enzyme, leading to the accumulation of tumor suppressor proteins and inducing cancer cell cycle arrest and apoptosis	AML MDS Lymphoma Solid tumors	TAK‐243 monotherapy	Phase 1	NCT03816319 NCT06223542
MLN4924	Pevonedistat	E1 NAE	Blocks the interaction between NAE and NEDDB	MDS/MPN Mesothelioma AML/MDS/CMML Lung cancer HR MDS Refractory ALL/NHL Myelofibrosis Intrahepatic cholangiocarcinoma Relapsed or refractory CLL or non‐Hodgkin lymphoma Recurrent or refractory solid tumors Melanoma MM	Pevonedistat+azacitidine Pevonedistat alone/+cisplatin Pevonedistat alone/+azacitidine Pevonedistat+docetaxel Pevonedistat+decitabine+cedazuridine Pevonedistat+VXLD Pevonedistat+ruxolitinib Pevonedistat+carboplatin+paclitaxel Pevonedistat+ibrutinib Pevonedistat+irinotecan+temozolomide Pevonedistat Pevonedistat+ixazomib	Phase 2 Phase 2 Phase 3 Phase 2 Phase2 Phase 1 Phase 1 Phase 2 Phase 1 Phase 1 Phase 1 Phase 1	NCT03238248 NCT03319537 NCT03268954 NCT03228186 NCT04985656 NCT03349281 NCT03386214 NCT04175912 NCT03479268 NCT03323034 NCT01011530 NCT03770260
NSC697923	/	E2 Ubc13	Inhibits the binding of Ubc13 to ubiquitin molecules and hinders the extension of the ubiquitin chain, exerting anti‐tumor effects through activation of the p53 and JNK pathways	Neuroblastoma Colorectal cancer Diffuse large B‐cell lymphoma (DLBCL) Melanoma	Mice model	Pre‐clinical	PMID: 24556694 PMID: 28476810 PMID: 22791293 PMID: 30224375
DHPO	/	E2 UbcH5c	DHPO directly binds to UbcH5c, affecting ubiquitination and inhibiting the NF‐κB signaling pathway to exert anti‐cancer effects	Pancreatic cancer	Mice model	Pre‐clinical	PMID: 35272681
RG7112	RO5045337	E3 MDM2	Occupies the p53‐binding pocket of MDM2, stabilizes p53 and activates the p53 pathway, leading to tumor cell cycle arrest and apoptosis	Hematologic neoplasms (myelogenous/lymphocytic leukemia) Advanced solid tumors	RO5045337 monotherapy	Phase 1	NCT00623870 NCT00559533
DS‐3032b	Milademetan	E3 MDM2	Targeted inhibition of MDM2 induces cell cycle arrest, senescence and apoptosis	MM AML Advanced solid tumors Lymphomas	Milademetan monotherapy Milademetan+quizartinib Milademetan+cytarabine+venetoclax Milademetan monotherapy	Phase 1 Phase 1 Phase 2 Phase 1	NCT02579824 NCT03552029 NCT03634228 NCT01877382
AMG 232	Navtemadlin KRT‐232	E3 MDM2	Selectively inhibits p53‐MDM2 interaction	MM AML Melanoma Soft tissue sarcoma Glioblastoma	KRT‐232+carfilzomib+lenalidomide+dexamethasone KRT‐232+cytarabine+idarubicin KRT‐232+trametinib+dabrafenib KRT‐232+radiotherapy KRT‐232+radiotherapy	Phase 1 Phase 1 Phase 1 Phase 1 Phase 1	NCT03031730 NCT04190550 NCT02110355 NCT03217266 NCT01723020
APG‐115	Alrizomadlin AA‐115	E3 MDM2	Blocks the interaction of MDM2 with p53 and induces cell cycle arrest and apoptosis in a p53‐dependent manner	AML, CMML, MDS Advanced solid tumor lymphoma BAP1 cancer syndrome Mesothelioma Melanoma Salivary gland carcinoma Neuroblastoma Liposarcoma Malignant peripheral nerve sheath tumors	APG‐115 alone/+azacitidine APG‐115 monotherapy APG‐115 monotherapy APG‐115 alone/+pembrolizumab APG‐115+carboplatin APG‐115 alone/+APG‐2575 APG‐115+toripalimab APG‐115+selumetinib	Phase 2 Phase 1 Phase 2 Phase 2 Phase 2 Phase 1 Phase 2 Phase 2	NCT04358393 NCT02935907 NCT06654050 NCT03611868 NCT03781986 NCT05701306 NCT04785196 NCT06735820
JNJ‐26854165	Serdemetan	E3 HDM2	HDM2 ubiquitin ligase antagonist that induces early apoptosis in p53 wild‐type cells	Advanced stage and/or refractory solid tumors	Serdemetan monotherapy	Phase 1	NCT00676910
CGM097	NVP‐CGM097	E3 HDM2	Inhibits HDM2‐p53 interaction	Solid tumors	CGM097 monotherapy	Phase 1	NCT01760525
SAR405838	MI‐77301	E3 HDM2	Inhibits HDM2‐p53 interaction and expresses anti‐tumor activity	Solid tumors	SAR405838+pimasertib	Phase 1	NCT01985191
CC 122	Avadomide	E3 celebron	Regulates cereblon E3 ligase activity, inhibits NF‐κB pathway, and induces apoptosis by blocking the cell cycle	DLBCL Melanoma	Avadomide+R‐CHOP Avadomide+nivolumab	Phase 1 phase 2	NCT03283202 NCT03834623
CC‐90009	Eragidomid	E3 celebron	Targets of GSPT1 for ubiquitination and proteasomal degradation	AML MDS/AML	CC‐90009+venetoclax+azacitidine+gilteritinib Escalating doses of CC‐90009	Phase 1 Phase 1	NCT04336982 NCT02848001
CC‐92480	Mezigdomide BMS‐986348	E3 celebron	Regulates cereblon E3 ligase activity	MM	CC‐92480+tazemetostat+dexamethasone	Phase 2	NCT06048250
ARV‐110	Bavdegalutamide	E3 AR	A specific androgen receptor (AR) PROTAC‐like degrader, promotes AR ubiquitination and degradation	Prostate cancer	ARV‐110 monotherapy	Phase 2	NCT03888612
ARV‐471	Vepdegestrant	E3 ER	An estrogen receptor PROTAC protein degrader, promotes the interaction between estrogen receptor alpha and the intracellular E3 ligase complex, leading to ubiquitination and subsequent degradation of estrogen receptors	Breast cancer	ARV‐471+palbociclib	Phase 2	NCT04072952

Abbreviations: ALL, acute lymphoblastic leukemia; AML, acute myeloid leukemia; CMML, chronic myelomonocytic leukemia; JNK, c‐Jun N‐terminal kinase; MM, multiple myeloma; MDS, myelodysplastic syndrome; MPN, myeloproliferative neoplasms; NHL, non‐Hodgkin's lymphoma.

*Source*: www.clinicaltrials.gov.

**TABLE 2:** Classification, mechanisms, targets and therapeutic applications of proteasome inhibitors.

Drug	Synonym	Target	Mechanisms	Cancer types	Treatments	Phase status	Clinical trial numbers
Bortezomib	Velcade PS‐341 LDP‐341 NSC681239	26S Proteasome (β5)	Inhibits proteasome‐mediated protein hydrolysis, disrupts the cell cycle, induces apoptosis and inhibits nuclear factor NF‐κB	MM Prostate cancer Ovarian cancer, peritoneal cancer, fallopian tube cancer MDS B‐cell lymphoma AML/ALL/CML Advanced refractory solid tumors, melanoma Cholangiocellular carcinoma Transitional cell carcinoma Breast cancer Thyroid cancer Lung cancer	Bortezomib+zarnestra Bortezomib+dexamethasone Bortezomib+hydroxychloroquine Bortezomib+docetaxel Bortezomib+platinum Bortezomib monotherapy Bortezomib monotherapy Bortezomib monotherapy Bortezomib+temozolomide Bortezomib monotherapy Bortezomib monotherapy Bortezomib+doxorubicin Bortezomib monotherapy Bortezomib monotherapy	Phase 2 Phase 2 Phase 1 Phase 2 Phase 1 Phase 2 Phase 2 Phase 1 Phase 2 Phase 3 Phase 2 Phase 2 Phase 2 Phase 2	NCT00361088 NCT00391157 NCT00568880 NCT00183937 NCT00059618 NCT00262873 NCT00038571 NCT00005064 NCT00512798 NCT03345303 NCT00072150 NCT00574236 NCT00104871 NCT00118144
Carfilzomib	PR‐171 Kyprolis	26S Proteasome (β5)		MM T‐cell lymphoma Mantle cell lymphoma ALL/AML Neuroendocrine cancers Advanced solid tumors Lung cancer	Carfilzomib monotherapy Carfilzomib monotherapy Carfilzomib monotherapy Carfilzomib monotherapy Carfilzomib monotherapy Carfilzomib+dexamethasone Carfilzomib+carboplatin+etoposide	Phase 2 Phase 2 Phase 2 Phase 1 Phase 1 Phase 1 Phase 1	NCT00511238 NCT01336920 NCT02042950 NCT01137747 NCT02318784 NCT02257476 NCT01987232
Ixazomib	Pevonedistat MLN4924	26S Proteasome (β5)		MM Follicular lymphoma Mantle cell lymphoma AML MDS Glioblastoma Advanced solid tumors	Ixazomib monotherapy Ixazomib monotherapy Ixazomib and ibrutinib Ixazomib monotherapy Ixazomib monotherapy Ixazomib monotherapy Ixazomib monotherapy	Phase 2 Phase 2 Phase 2 Phase 2 Phase 2 Phase 1 Phase 1	NCT00963820 NCT01939899 NCT03323151 NCT02030405 NCT02302846 NCT02630030 NCT01912222

Abbreviations: ALL, acute lymphoblastic leukemia; AML, acute myeloid leukemia; CMML, chronic myelomonocytic leukemia; MDS, myelodysplastic syndrome; MM, multiple myeloma; NHL, non‐Hodgkin's lymphoma.

*Source*: www.clinicaltrials.gov.

#### 4.1.1 Ubiquitin‐Activating Enzyme E1

The initial UPS enzyme, E1, is a crucial component of the UPS. It catalyzes the activation of ubiquitin molecules [71]. The multi‐domain structure of classic E1 includes the pseudo‐dimeric adenylate domain involved in Ubl activation, the Cys domain that carries catalytic cysteine residues, and the ubiquitin folding domain of the E1 enzyme involved in E2 recruitment [72]. The cysteine domain serves as the active center and plays a crucial role in ubiquitin transfer [73]. The subtypes of E1 enzymes include E1A and E2B, of which E1A is present in yeast and E1B is expressed in mammals. Currently, only UBA1 and UBA6 (ubiquitin‐activating enzymes) are found in mammalian cells. UBA1 is the main ubiquitin‐activating enzyme that participates in the ubiquitination of most proteins in cells [74]. It is a monomeric E1 enzyme, and there is relatively little research on UBA6 [75]. In various tumors, the expression of ubiquitin‐activating enzymes is often abnormally elevated, which is closely related to their critical role in the UPS. Ubiquitin‐activating enzymes participate in various biological processes within cells by catalyzing the ubiquitination of target proteins. The onset and progression of cancers are significantly influenced by the aberrant expression and function of ubiquitin‐activating enzymes [76]. Tumor cells have increased dependence on protein ubiquitination; therefore, targeted inhibition of the ubiquitin‐activating enzyme E1 may be a therapeutic strategy for cancers [77]. In response to these mechanisms, researchers have developed various targeted therapeutic drugs to inhibit the expression or function of E1, thereby controlling cancer development. These drugs include small‐molecule inhibitors (Table 1).

PYR‐41 is the first reported effective UBE1 inhibitor that can penetrate cells. The intramolecular nitro group reacts with the cysteine thiol group of CCD to irreversibly inhibit UBA1 activity [49]. It inhibits the formation of thioester bonds between UBA1 and Ub without affecting the transfer of Ub to E2 via UBA1‐carrying ubiquitin molecules [78]. PYR‐41 simultaneously inhibits degradation and non‐degradation forms of ubiquitination, as well as cytokine‐induced activation of NF‐kB, stabilizing p53 and increasing its transcriptional activity [79]. The mechanism of action of PYZD‐4409 is similar to that of PYR‐41, which stabilizes p53 and cyclin D3 and induces endoplasmic reticulum stress, leading to cell death. JS‐K exhibits anti‐angiogenic effects in MM models and can induce DNA double‐strand breaks, thereby activating DNA damage [73].

#### 4.1.2 Ubiquitin‐Conjugating Enzyme E2

More than 40 E2 enzymes have been identified in mammalian cells, most of which contain ubiquitin‐binding domains [80]. Some E2 enzymes are composed only of ubiquitin C (UBC) domains, whereas others have typical short extensions of unstructured regions at one or both ends [81]. Ubiquitin molecules activated by the E1 enzyme can form E2 Ub complexes with the E2 enzyme through thioester bonds and then bind to different E3 ligases. Under E3 ligase catalysis, ubiquitin molecules are transferred to substrate proteins that serve as the central enzymes for ubiquitination. E2 determines the topological process of ubiquitination and is responsible for recruiting different E3 ligases [82]. Due to the low levels of ubiquitin‐activating enzyme E1, inhibiting E1 results in the inhibition of most ubiquitination processes with poor selectivity. Therefore, anti‐cancer drugs targeting the E2 enzyme can provide specificity that E1 enzyme inhibitors cannot achieve.

UBC3 (CDC340) is a ubiquitin‐binding enzyme. It modifies target proteins via polyubiquitination and regulates proteins involved in cell cycle regulation, such as p27. UBC3 (CDC34) is phosphorylated in vivo, providing another potential anti‐tumor activity [83]. CDC34 is highly expressed in MM cells, and studies have shown that blocking CDC34 enhances its anti‐myeloma effects. Furthermore, CDC34 is inhibited by CC0651, and combining CC0651 with regularly used anti‐MM medications is a promising new therapeutic method [83, 84].

In recent years, small‐molecule inhibitors targeting E2 enzymes have demonstrated significant anti‐tumor potential, with NSC697923 and DHPO as representative compounds that offer new strategies for tumor‐targeted therapy [85] (Table 1).

NSC697923 is a selective UBE2N (Ubc13) inhibitor that induces apoptosis in tumor cells through a dual mechanism: In p53 wild‐type neuroblastoma, it promotes the activation of pro‐apoptotic genes by nuclear translocation of p53; and in p53 mutant cells, it triggers the mitochondrial apoptotic pathway by activating the c‐Jun N‐terminal kinase (JNK)/MAPK pathway [86]. A report showed that NSC697923 significantly inhibited neuroblastoma xenograft tumor growth and blocked the constitutive NF‐κB signaling pathway in diffuse large B‐cell lymphoma (DLBCL), thereby inhibiting tumor proliferation [87]. The naturally occurring small molecule DHPO, a UbcH5c‐specific inhibitor, significantly downregulated the expression of anti‐apoptotic genes (e.g., c‐Myc and Mcl‐1) in pancreatic cancer cells by blocking the ubiquitinated degradation of IκBα and sustained inhibition of NF‐κB nuclear translocation [88].

#### 4.1.3 Ubiquitin Ligases E3

The ability of E3 ligases to selectively recognize substrate proteins and catalyze the transfer of ubiquitin from E2‐ubiquitin complexes to these substrates is essential for the ubiquitin–proteasome degradation pathway [89]. This selective ubiquitination ensures the specificity of the process. The human genome encodes approximately 600 distinct types of ubiquitin ligase E3, which are primarily classified based on their structures and the mechanisms of ubiquitin transfer [90]. Three primary categories of E3 ligases—Really Interesting New Gene (RING), akin to the E6AP carboxyl terminus (HECT), and RING‐between‐RING (RBR)—show distinct structural and molecular characteristics, paving the way for identifying two primary routes for ubiquitin transfer. HECT and RBR E3 ligases must first accept the E2‐ubiquitin complex before transferring it to the substrate. Conversely, RING‐type E3 ligases can directly facilitate the movement of ubiquitin from the E2‐ubiquitin complex to the substrate [91]. The RING E3 ligase family, which includes both the U‐box and RING domains, represents the largest superfamily of ubiquitin E3 ligases. Despite their structural similarities, the U‐box and RING domains differ in their ability to bind zinc ions; the former does not, whereas the latter does. RING E3 ligase functions as an intermediary, transferring activated ubiquitin from E2 to the substrate protein without directly binding to the ubiquitin molecule. The HECT E3 ligase, on the other hand, possesses a HECT domain with a conserved cysteine residue that forms a thioester bond with the ubiquitin carried by E2, subsequently transferring ubiquitin to the substrate. Meanwhile, the RBR E3 ligase is characterized by the presence of an RBR domain, which is divided by the “in‐between‐RING” domain [92]. RING2 has catalytic cysteine residues that can form thioester bonds with ubiquitin and pass it to target proteins, whereas RING1 binds to the E2‐ubiquitin complex [93]. Although the lack of most E3 ubiquitin ligases may not cause a major increase in malignant hematological illnesses, they can impair hematopoietic stem cell capacity or alter the direction of lineage differentiation [94]. The activity of ubiquitin ligases is abnormally frequent. Specific ubiquitin ligases promote the survival and proliferation of myeloma cells, making them resistant to chemotherapeutic drugs [95]. A deeper understanding of ubiquitin ligases may provide novel insights to solve these problems [96].

Immunomodulatory drugs (IMiDs) are similar to thalidomide, which is a glutamate derivative owing to its anti‐tumor necrosis factor (TNF)‐α activity, anti‐angiogenic properties, and strong anti‐inflammatory effects [97]. There are currently three types of IMiDs that can be used to treat myeloma: thalidomide, lenalidomide, and pomalidomide.

**FIGURE 2:**
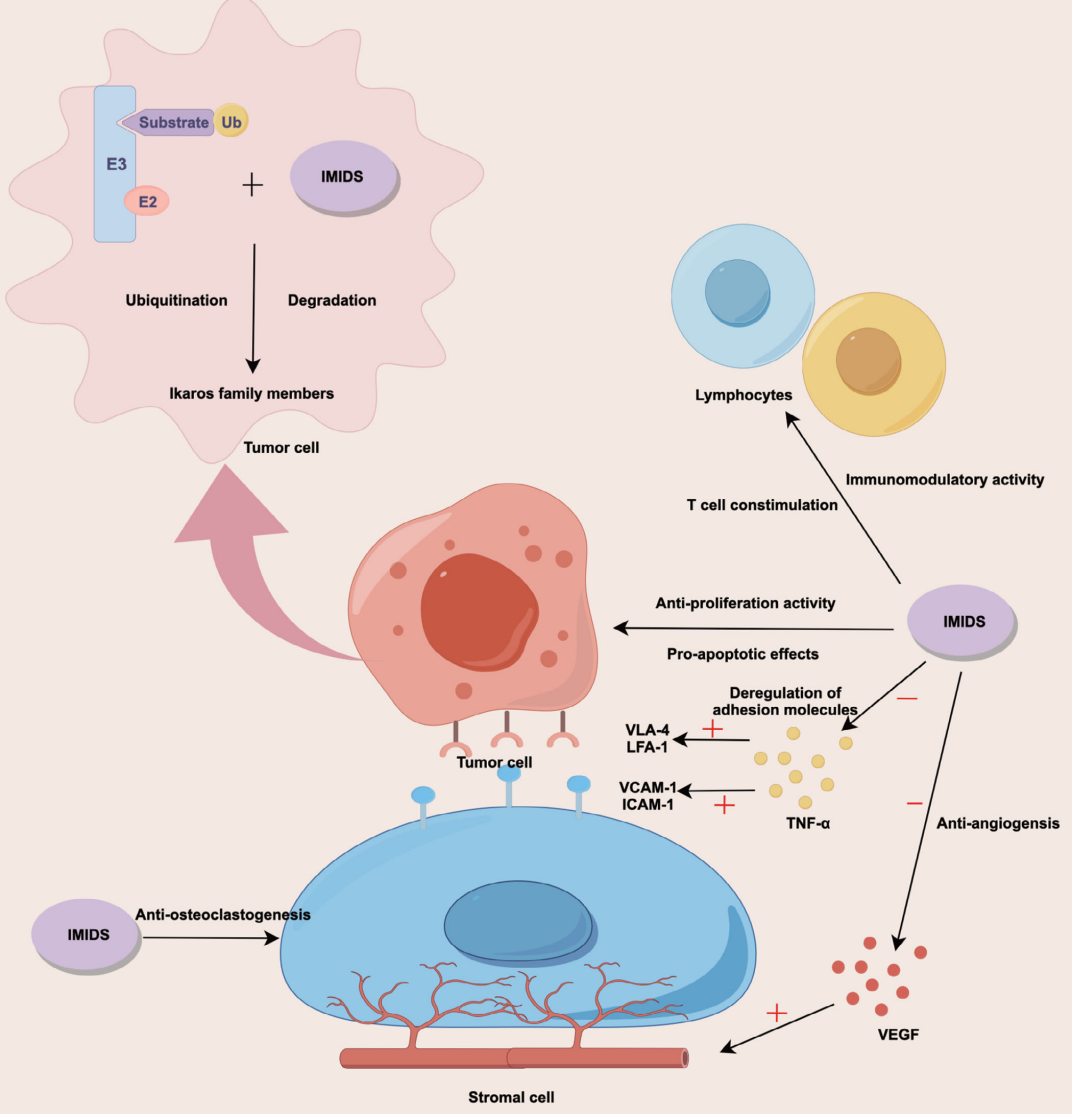
This figure shows the mechanism of immunomodulatory drugs in the treatment of cancer, including immunomodulatory effects through NK/T cell activation and Treg suppression to enhance anti‐tumor immunity; direct pro‐apoptotic actions via cyclin‐dependent kinase inhibition and Fas‐mediated apoptosis; suppression of TNF‐α‐induced adhesion molecules (e.g., ICAM‐1, VCAM‐1) to disrupt malignant cell interactions and overcome drug resistance; potent anti‐angiogenic activity via VEGF reduction, particularly with thalidomide; and anti‐osteoclastogenic effects through the inhibition of osteoclast differentiation to mitigate bone destruction.

The anti‐tumor effect of IMiDs is mainly achieved through the following aspects (Figure 2). First, the immunomodulatory effect is significant, as various immune cells and other cell types contribute to the development and progression of tumor [98]. These cells include mesenchymal stem cells, stromal and endothelial cells, tumor‐associated fibroblasts, and immune cells such as dendritic cells, macrophages, helper T cells, and regulatory T cells (Tregs). This diverse cellular environment plays a critical role in the pathogenesis of tumor [99]. Upon direct contact with tumor cells, these cells secrete extracellular vesicles and diverse cytokines, including IL‐6, IL‐10, and vascular endothelial growth factor (VEGF). They can also activate different signal transduction pathways, which in turn regulate tumor cell aggregation, proliferation, survival, angiogenesis, drug resistance, and ultimately the occurrence and development of tumor. The secretion of cytokines, particularly IL‐6, is the main regulatory mechanism. Anti‐tumor immune cells include natural killer (NK), NK T, and T cells, which produce interferon‐γ through indirect regulation by cytokines or direct regulation by receptor ligand binding [100]. The growth, survival, angiogenesis, and drug resistance of tumor cells are regulated to exert anti‐tumor effects. Cytokine production is the main mechanism by which tumor growth is inhibited. IMiDs strengthen the immune response against tumor by activating both T and NK cells [101]. In addition, they inhibit the activity of Tregs, which can enhance tumor‐specific immunity and improve control over tumor growth.

Second, IMiDs exert anti‐inflammatory effects by suppressing various cytokines that possess both pro‐inflammatory and pro‐tumor properties [102]. Cyclooxygenase‐2 (COX‐2), a key enzyme in the development of multiple malignancies, catalyzes the conversion of arachidonic acid into several pro‐inflammatory prostaglandins, including PGE2, which promotes tumor angiogenesis and IL‐6 production [103]. Consequently, COX‐2 inhibitors may offer therapeutic benefits for MM by inducing apoptosis in myeloma cells and reducing COX‐2 expression in activated peripheral blood mononuclear cells, although they do not affect the expression of COX‐1 [104].

Third, regarding anti‐angiogenic effects, microvasculature formation is linked to the development of tumor, and all IMiDs exhibit anti‐angiogenic activity [105]. Thalidomide has stronger anti‐angiogenic activity than lenalidomide and pomalidomide, which, in turn, have stronger immune‐boosting properties than thalidomide [106]. Fourth, by inhibiting adhesion molecules, TNF‐α can upregulate adhesion molecules that promote plasma cell–bone marrow mesenchymal cell interactions [107]. In contrast, IMiDs can inhibit TNF‐α, thus inhibiting the expression of adhesion molecules on the surface of plasma cells and bone marrow mesenchymal cells, such as ICAM‐1, VLA‐4, LFA‐1, and VCAM‐1 [42, 108]. Therefore, IMiDs can overcome malignant PC cell adhesion‐mediated drug resistance. Fifth, IMiDs may have anti‐osteoclastogenic properties despite the lack of substantial clinical evidence supporting their preventive benefit against tumor‐related bone disorders. In fact, IMiDs directly and dose‐dependently block osteoclast development in vitro [109].

Last, regarding their anti‐tumor cell proliferation effect, IMiDs can exert anti‐plasma cell proliferation effects by inhibiting CDKs, activating Fas‐mediated cell death, downregulating anti‐apoptotic proteins, and other pathways [110, 111]. Thalidomide is an angiogenesis inhibitor and immune modulator with anti‐tumor activity against tumor; anti‐angiogenic effects can reduce the blood concentration of VEGF, thereby reducing the blood supply to tumor cells [112]. This not only acts on tumor cells and stromal cells but also inhibits their proliferation and regulates cell surface adhesion molecules, thereby altering the survival of tumor cells, affecting the secretion of various cytokines in tumor and stromal cells, altering their biological activity, and acting on T cells to exert immune regulatory effects [48]. The main adverse reactions include drowsiness, constipation, loneliness, peripheral neuritis, dizziness, fatigue, and decreased white blood cell count [113].

Thalidomide has no bone marrow toxicity, and the combined vincristine, doxorubicin, and dexamethasone regimen for treating cancer has advantages such as fewer side effects, good tolerance, convenient administration, and significant therapeutic effects [53]. Lenalidomide is an immunomodulatory derivative of thalidomide with strong anti‐myeloma activity. Its toxicity is similar to that of thalidomide. However, typical adverse reactions such as drowsiness, constipation, and peripheral neuropathy are not prominent [114]. A significant adverse reaction to lenalidomide is the induction of cell reduction, mainly neutropenia and thrombocytopenia, with less anemia. Lenalidomide is effective in patients with newly diagnosed, relapsed, or refractory MM [115].

Pomalidomide is a third‐generation immune modulator that exerts anti‐tumor and immunoregulatory effects by binding to cereblon targets [116]. It also exerts anti‐tumor effects by inhibiting angiogenesis and affecting the bone marrow microenvironment [117]. Early adverse reactions mainly include hematological adverse reactions and pulmonary infections, which can be managed with hematopoietic drugs and antibiotics [118]. A small number of patients may experience peripheral neuropathy, fatigue, diarrhea, and no thrombosis. Daratumumab, low‐dose dexamethasone, and pomalidomide have been demonstrated to be safe and effective therapeutic options for patients with relapsed/refractory MM after disease progression or lenalidomide use [111, 119]. In summary, the pomalidomide‐based regimen has significant short‐term efficacy in high‐risk patients with MM and is effective in all subgroups, including initial onset, recurrent refractory disease, renal dysfunction, and high‐risk cytogenetics [120].

#### 4.1.4 Protein Hydrolysis‐Targeting Chimeras

**FIGURE 3:**
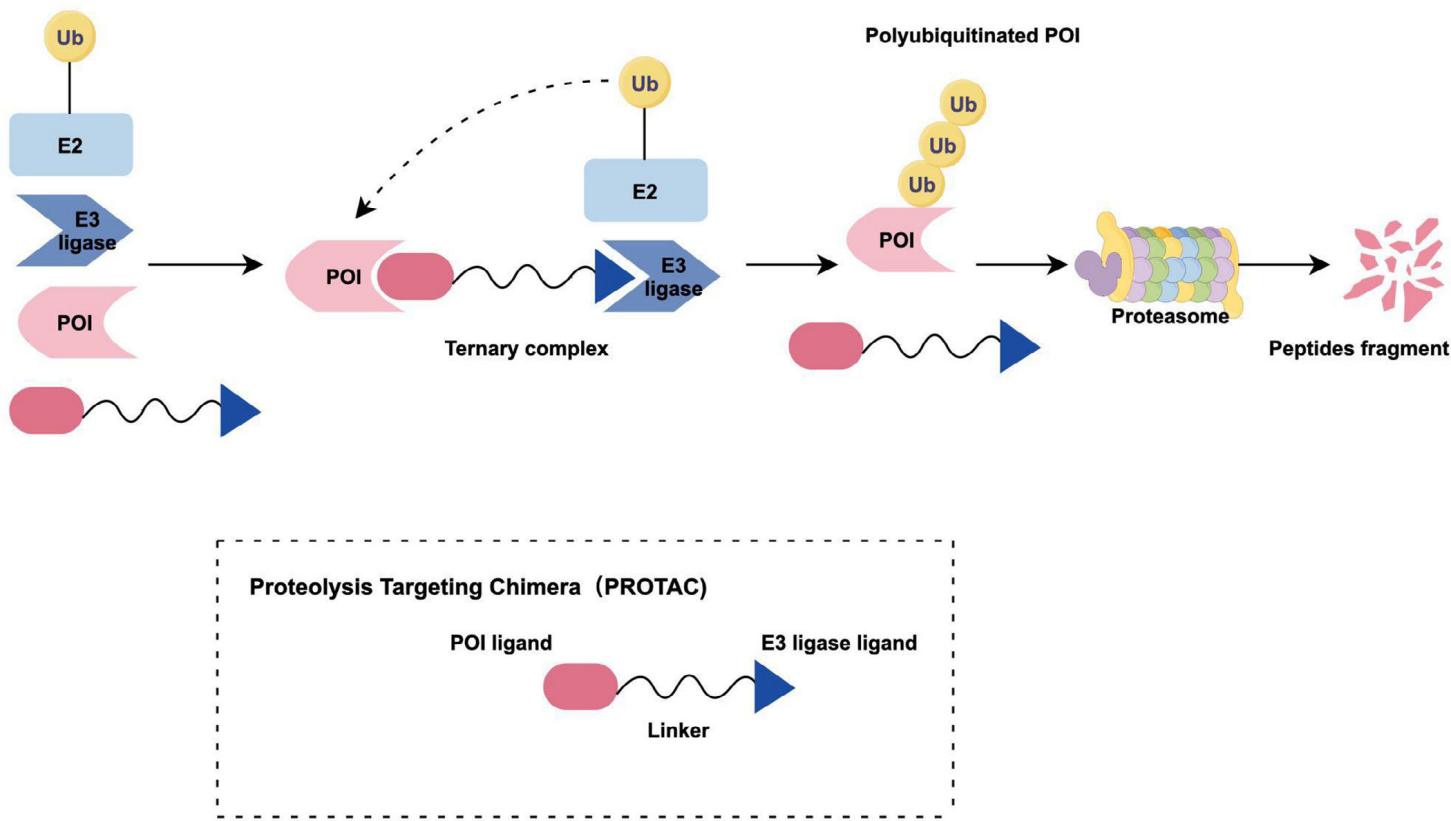
This figure shows the structure and working mechanism of PROTAC: A PROTAC molecule is composed of a POI ligand (typically a small‐molecule inhibitor) and an E3 ubiquitin ligase ligand, covalently connected by a linker (mostly 5 ‐ 15 carbon or other atoms). It induces proximity between ligases and substrates, enabling a single PROTAC molecule to repeatedly catalyze ubiquitination reactions, forming ubiquitin chains on the substrate, which are then recognized by proteasomes for degradation of the target protein into small peptides.

PROTACs are a class of heterologous bifunctional molecules that can degrade certain proteins using intracellular waste‐sorting systems [121]. A PROTAC is composed of three key components: an E3 ubiquitin ligase‐recruiting element [122], a ligand that binds to the target protein at the opposite end, and a linker that connects these two ligands in the center [123]. PROTACs initiate degradation cascade reactions by forming a ternary complex with E3 and targeting proteins, bringing the ubiquitination system closer to the subsequent targeting of protein ubiquitination [124]. Once the target proteins are polyubiquitinated, they are recognized by the 26S proteasome and subsequently degraded [125] (Figure 3).

PROTACs target many non‐pharmacological disease‐related proteins [126]. They have good targeting and efficient degradation properties and show good prospects in the field of malignant tumor treatment [127]. ARV‐825, which consists of pomalidomide and the BRD4 inhibitor OTX015, causes degradation of BRD4 and suppression of sub‐nanomolar levels of Burkitt lymphoma cell proliferation [128]. Owing to its excellent targeting and effective degradation capabilities, it has shown great promise for the treatment of malignant tumors. Pomalidomide and the BRD4 inhibitor OTX015 together form ARV‐825, which inhibits BRD4 and prevents the growth of Burkitt's lymphoma cells [129]. This also shows significant BRD4 degradation activity and suppresses the proliferation of secondary acute myeloid leukemia and MM cells.

#### 4.1.5 Molecular Glue

Small molecules known as “molecular glue” can create new contact between target proteins and E3 ubiquitin ligase substrate receptors [130], which in turn causes the degradation of the target protein [131]. Molecular gel‐degrading agents such as PROTACs are potential therapeutic candidates for cancer treatment because they can drastically reduce the demand for activity‐related pockets on target proteins [132]. Molecular glue promotes the ubiquitination of proteins of interest by enhancing protein–protein interactions between ligases and potential substrates [133].

### 4.2 Treatment of Cancer Based on Proteasome System

In approximately 1.8% of all cancers, MM is the second most prevalent hematological malignancy in the United States [134]. Although it is often considered incurable, the survival rate of patients with myeloma is increasing annually with improvements in medical technology [135]. Proteasome inhibitors are currently one of the best options for treating myeloma and have achieved significant results in the current treatment progress thereof. Proteasome inhibitors have a powerful effect on myeloma treatment because myeloma cells are dependent on proteasomes [136]. Proteasomes are considered “garbage treatment plants” that degrade proteins into peptides or amino acids. Tumor cells are more metabolically active than normal cells and require proteasomes to process and break down proteins in order to prevent excessive protein accumulation or cytotoxicity [137].

The US Food and Drug Administration has approved ixazomib, oprozomib, carfilzomib, bortezomib, and carfilzomib as proteasome inhibitors for cancer treatment [138]. Tumor cells eventually perish owing to the biological features of the disease [139]. Thus, proteasome complexes are especially important in tumor cells to prevent excess protein and metabolite accumulation. Therefore, through targeted mechanisms, proteasome inhibitors can selectively induce apoptosis in tumor cells, thereby controlling the disease [140].

#### 4.2.1 26S Proteasome

**FIGURE 4:**
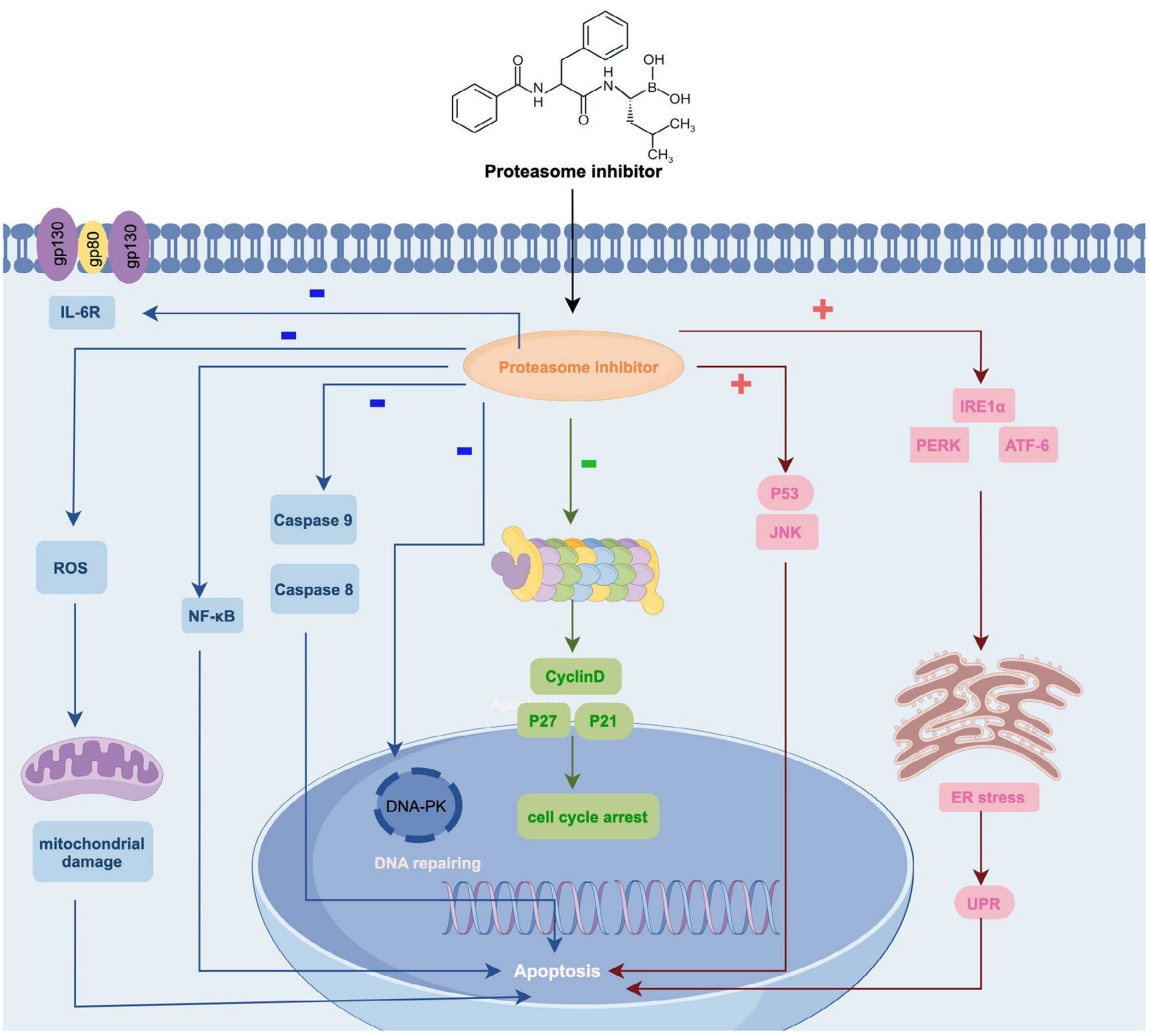
This figure shows that the structure and functional mechanisms of the 26S proteasome in myeloma: The 20S core particle comprises four stacked rings: two outer α rings (α1‐7 subunits) and two inner β rings (β1‐7 subunits). Proteasome inhibitors exert anti‐tumor effects through: (1) NF‐κB pathway inhibition, (2) JNK/p53‐mediated apoptosis, (3) stabilization of pro‐apoptotic proteins (Bim/BIK/BID/Bax), and (4) cell cycle disruption via cyclin accumulation.

In eukaryotes, the main proteasome, known as the 26S proteasome, plays a crucial role in decomposing proteins found on the surfaces of numerous organelles, cytoplasm, and nucleus [141]. The 26S proteasome, composed of 20SCP and 19SRP, is a massive protein complex with approximately 50 subunits [38, 142]. Its function is to degrade proteins into peptides and has high specificity and sensitivity for the decomposition of proteins [143] (Figure 4). The 20S core granules (CP) of the 26S proteasome hydrolyze unfolded peptides into short peptides or amino acids, whereas 19S regulatory granules (RP) detect degradation signals and unfold target protein substrates [141]. Two 19S regulatory particles join a central 20S particle and a central 20S CP to form this subcomplex [144]. The entity responsible for protein degradation is 20SCP; it is the disintegration apparatus of the proteasome that breaks down proteins into peptides [145]. The 19S RP regulator functions as a receptor for polyubiquitinated proteins and controls the breakdown of ubiquitin‐tagged substrates by enabling translocation of the 20S CP into the catalytic chamber in an ATP‐dependent manner [146]. The fact that many 19S subunit components participate in ubiquitin ligase or deubiquitinated E3 activity suggests that the destiny of substrates tagged with ubiquitin is dynamically controlled [147]. In conclusion, the 26S proteasome is an important protein in myeloma cells that is primarily responsible for protein degradation in the cytoplasm and nucleus [148]. Proteins that need to be metabolized in tumor cells are targeted and transported to the proteasome under the action of ubiquitin, recognized by 19SRP and explained by 20SCP, and finally the decomposed proteins become polypeptides [149].

#### 4.2.2 Substrate Requirements for Proteasome Degradation

Although proteasomes process and degrade many proteins in cells, they must have two important functions: targeted signaling and unstructured initiation regions. Targeted signaling allows the proteasome to precisely locate specific proteins and recognize and degrade them [150]. The unstructured starting region is not only necessary for the translocation mechanism to reach the depth of the central pore of the proteasome but also plays an important role in involving substrates in proteasome processing [151]. This step is crucial for the direct coupling between protein substrate degradation and proteasomal de‐ubiquitination. The sequence composition of this region has a significant effect on the rate of substrate degradation in vivo and in vitro [152].

#### 4.2.3 Proteasome Inhibitors

##### 4.2.3.1 Bortezomib

**FIGURE 5:**
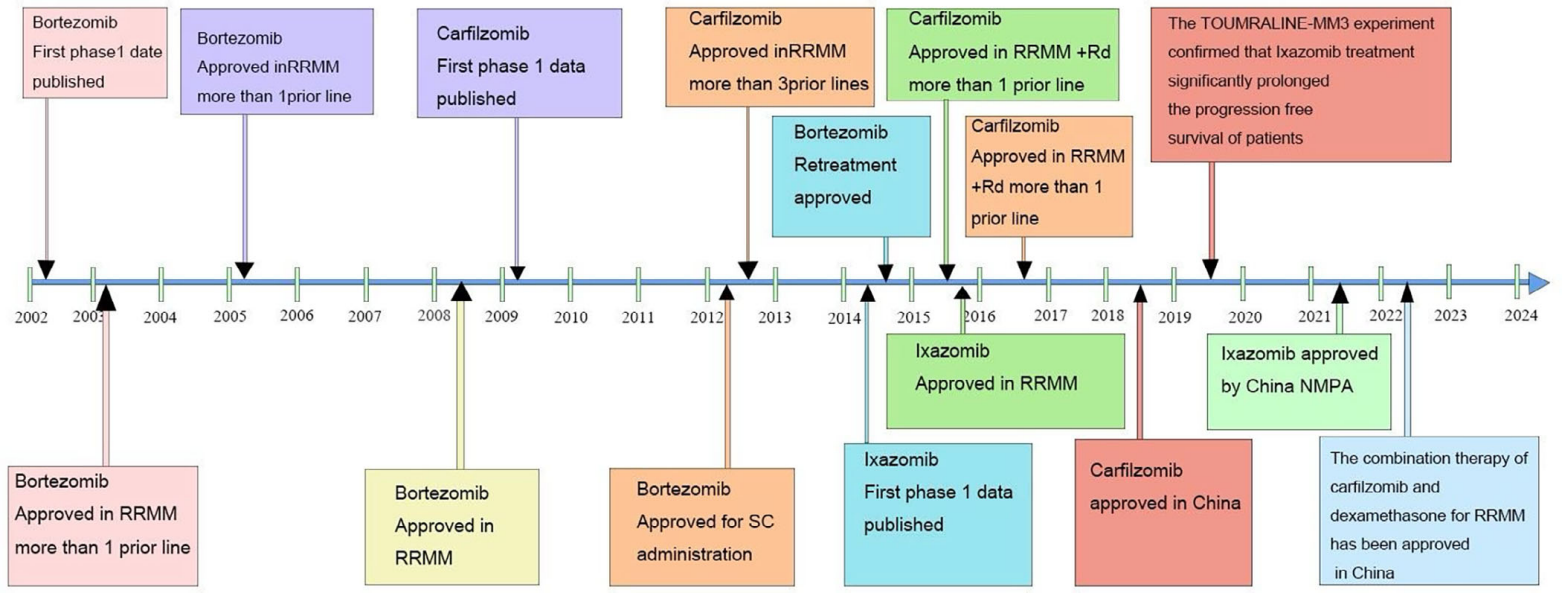
This timeline chart illustrates the developmental milestones of proteasome inhibitors from 2002 to 2024 (taking myeloma as an example). It tracks key events like the publication of first—Phase 1 data (e.g., bortezomib in 2003, carfilzomib in 2009) and regulatory approvals for various indications and administration routes (such as bortezomib's approval in RRMM and for SC administration). Ixazomib also has its Phase 1 data release and approval milestones shown, while the TOUMRALINE—MM3 experiment result about ixazomib's impact on progression—free survival is included. Overall, it maps the evolving application and approval landscape of these inhibitors in treating relapsed/refractory multiple myeloma (RRMM) over two decades.

In 2003, the FDA approved bortezomib as the first proteasome inhibitor for treating MM (Figure 5) [153]. Known chemically as [3‐methyl‐1‐(3‐phenyl‐2‐pyrazin‐2‐ylcarbonylamino‐propanoyl) amino‐butyl] boronic acid, bortezomib's IUPAC formula is C19H25BN4O4. Acting as a reversible inhibitor of the 26S proteasome [154], bortezomib (BTZ) plays a critical role in disrupting the protein degradation process [155]. The boronic acid group of this entity attaches to the threonine hydroxyl group in the β5‐subunit's active site, thereby hindering the proteasome's activity similar to chymotrypsin. This interaction makes BTZ a unique reversible inhibitor of the 20S CP β‐subunit associated with the 26S proteasome [156]. The incomplete and transient inhibition of the 26S proteasome by bortezomib triggers apoptosis in myeloma cells, largely through the activation of BTZ‐8/9 [157]. Additionally, bortezomib blocks the NF‐κB signaling pathway, reducing the expression of antiapoptotic genes under its control [158]. In various tumor cell lines lacking p53 signaling, bortezomib has been shown to induce the expression of NOXA, a pro‐apoptotic protein, thereby inhibiting tumor growth. Interestingly, BTZ selectively induces NOXA expression 20 to 60 times higher in cancer cells, compared to normal cells. This selective induction is dependent on the c‐Myc binding site in the NOXA promoter, and when c‐Myc is depleted, BTZ's ability to induce NOXA in tumor cells is significantly reduced [159]. However, bortezomib's therapeutic use is constrained by its off‐target effects, drug resistance, and toxicities associated with proteasomal inhibition. Binding to subunit 5 inhibits cholinergic protease activity, causing an accumulation of cytotoxic proteins that cannot be properly degraded, ultimately leading to cytotoxicity [160]. Various side effects have been documented with bortezomib use, including myelosuppression, fatigue, pain, and peripheral neuropathy, all of which vary depending on the dosage administered [161]. Moreover, approximately 60% of patients develop resistance to BTZ over time. Addressing this matter, the FDA has approved integrating bortezomib with chemotherapy medications like doxorubicin and dexamethasone in clinical trials, targeting to enhance its efficacy and combat drug resistance [162].

##### 4.2.3.2 Carfilzomib

Carfilzomib, approved by the US FDA in 2012 as the second proteasome inhibitor [163], is used for treating MM patients who have developed resistance to bortezomib [164]. It exhibits good systemic distribution, though it is unable to penetrate the blood–brain barrier [165]. With a short half‐life of approximately 30 min, carfilzomib undergoes extrahepatic metabolism into inactive metabolites, making it independent of liver function [166]. This significantly reduces the likelihood of interactions with other medications metabolized by the liver, contrasting with bortezomib, which is primarily cleared through hepatic pathways [167]. It covalently binds to the threonine residue at the proteasome's active site, using its epoxide group as the reactive agent. This covalent and irreversible interaction allows carfilzomib to inhibit proteasome activity more efficiently than bortezomib, particularly targeting the chymotrypsin‐like proteasome and immunoproteasome activities, mainly through the L5 subunit [165]. In MM models, carfilzomib has demonstrated potent binding and inhibition of these activities, leading to the accumulation of ubiquitinated proteins and resulting in a dose‐ and time‐dependent suppression of cell proliferation, eventually triggering apoptosis [168]. The process of programmed cell death triggered by Carfilzomib includes triggering JNK, depolarizing the mitochondrial membrane, releasing cytochrome c, and activating both internal and external caspase pathways. This agent also inhibits proliferation and induces apoptosis in patient‐derived MM cells as well as in neoplastic cells from patients with other hematologic malignancies [169]. Moreover, carfilzomib offers a more selective mechanism of action, higher chemical stability, and fewer adverse effects compared to bortezomib. Carfilzomib, owing to its low solubility in water, is not suitable for oral administration and should be administered intravenously [170]. Its combination with lenalidomide and dexamethasone has proven to markedly enhance overall and progression‐free survival rates in individuals with recurrent and resistant MM [171]. As a potent proteasome inhibitor, carfilzomib is an effective therapy with a favorable side effect profile, including a low incidence of peripheral neuropathy [172]. In MM treatment, carfilzomib represents a valuable addition to therapeutic options, complementing agents like bortezomib, alkylators, corticosteroids, and IMiDs such as thalidomide and lenalidomide [173]. The safety profile of carfilzomib has been deemed favorable, with no significant long‐term complications reported. The most common adverse reactions include anemia, diarrhea, nausea, vomiting, thrombocytopenia, and neutropenia. Fatigue and nausea are the most frequently reported non‐hematological side effects [174], while neutropenia and thrombocytopenia are the primary grade 3/4 toxicities [175].

##### 4.2.3.3 Ixazomib

The US FDA sanctioned ixazomib in 2015 as the inaugural proteasome inhibitor to be taken orally for MM treatment [176]. Ixazomib works by binding to catalytic proteins, inhibiting their activity. Upon entering the bloodstream, ixazomib rapidly converts from a prodrug into its active form [177]. Its active state contains boric acid groups, which function as reversible inhibitors. Ixazomib is an attractive therapeutic option due to its oral administration, offering convenience to patients [178]. This medication is recommended for MM patients with at least one previous treatment and is frequently mixed with lenalidomide and dexamethasone [179]. Ixazomib acts as a specific, powerful, and reversible blocker of the 20S proteasome, favoring the β5 chymotrypsin‐like proteolytic site. Although it also inhibits the β1 caspase‐like and β2 trypsin‐like sites, its primary activity is on the β5 site. Ixazomib is rapidly absorbed, with peak plasma concentrations reached approximately 1 h after administration. However, a high‐fat meal can significantly reduce both the rate and extent of ixazomib absorption, supporting the recommendation for fasting before intake [180]. Common side effects of ixazomib include gastrointestinal issues like diarrhea, constipation, and nausea, as well as hematologic effects such as cytopenias, peripheral neuropathy, peripheral edema, vomiting, and back pain [181]. In conclusion, ixazomib is a promising proteasome inhibitor for the treatment of MM, particularly when used in combination with lenalidomide and dexamethasone after prior therapies [182]. Compared to bortezomib, a first‐generation proteasome inhibitor, ixazomib has a more favorable side effect profile and offers the added advantage of oral administration, enhancing its clinical utility [136].

##### 4.2.3.4 Oprozomib

In preclinical studies, the tripeptide analogue of carfilzomib, oprozomib, demonstrated anti‐cancer efficacy similar to that of carfilzomib [183]. Oprozomib has demonstrated encouraging efficacy in patients with RRMM, either alone or in combination [184]. Continuous/extended therapy, which includes all‐oral regimens, is becoming increasingly important in the treatment of newly diagnosed MM (NDMM) and needs to be conveniently administered [185]. Owing to the growing significance of continuous/extended therapy for NDMM, treatments, such as all‐oral regimens, must be simple to administer. Oprozomib is anticipated to be a practical therapeutic choice, particularly for oral regimens. Oprozomib, a novel irreversible proteasome inhibitor, is a tripeptide epoxide that is an orally bioavailable analogue of carfilzomib [186]. Oprozomib is a novel, bioavailable, and orally administered proteasome inhibitor, which can inhibit bortezomib‐resistant myeloma cells and induce apoptosis, which is a major advantage in myeloma treatment [184]. Oprozomib inhibits MM cell migration and, like bortezomib, oprozomib inhibits proteasome chymotrypsin‐like activity. Oprozomib, as an oral PI drug, has been studied for its intestinal absorption properties in preclinical species. Oprozomib cytotoxicity occurs concomitantly with the activation of caspase‐8, caspase‐9, caspase‐3, and poly(ADP)ribose polymerase. So, the adverse reactions it mainly causes are also related to gastrointestinal reactions, which mainly cause nausea and vomiting, and abdominal discomfort [187].

#### 4.2.4 Combination Therapy for MM

##### 4.2.4.1 Combination therapy with proteasome inhibitors and IMiDs

Early Phase 2 studies on bortezomib for the treatment of MM have shown that the addition of dexamethasone can enhance the pharmacological activity of bortezomib, reflecting the synergistic advantage of proteasome inhibition and corticosteroids in the treatment of myeloma [188]. With advancements in clinical technology, this combination therapy has largely replaced single‐dose bortezomib. Based on survival benefits, compared to the combination of melphalan and prednisone, the combination of melphalan, prednisone, and thalidomide and the combination of melphalan, prednisone, and bortezomib are the preferred first‐line therapies, if available and well tolerated. For elderly MM patients, two separate studies have shown the efficacy of lenalidomide and dexamethasone as a combined treatment [189]. The use of proteasome inhibitors, IMiDS (thalidomide, lenalidomide, or pomalidomide), along with dexamethasone, is recognized in clinical settings as a potent therapy for myeloma, extensively researched and applied in the creation of all three sanctioned proteasome inhibitors. The trio of bortezomib, thalidomide, and dexamethasone (VTD) marked the initial use of both an IMiD and a proteasome inhibitor. The combination treatment regimen greatly reduces the likelihood of peripheral neuropathy [190].

##### 4.2.4.2 Combination Therapy with Proteasome Inhibitors and Conventional Chemotherapy

One important reason why proteasome inhibitors stand out in the treatment of myeloma is that they exhibit synergistic effects and enhance clinical activity with traditional chemotherapeutic drugs (including alkylating agents and anthracycline drugs), which are suitable for the early treatment of myeloma. For treating MM, merging carfilzomib, lenalidomide, and dexamethasone can markedly enhance patient survival rates [191]. A significant benefit in progression‐free survival with the addition of ixazomib to carfilzomib and dexamethasone in patients with relapsed MM, compared with carfilzomib and dexamethasone alone has been observed. Treatment with ixazomib, lenalidomide, and dexamethasone is a viable option for transplant‐ineligible patients with NDMM who benefit from an all‐oral triple therapy regimen [115]. Carfilzomib with IMiDs for the treatment of NDMM [192]. In addition, several other carfilzomib‐based three‐drug regimens such as carfilzomib, cyclophosphamide, and dexamethasone and carfilzomib, pomalidomide, and dexamethasone have been evaluated in RRMM [193].

## 5 Summary and Future Outlooks

The ubiquitin proteasome framework holds potential for advancements in cancer clinical practice, and this analysis thoroughly explains its influence on managing tumors [194]. The UPS is composed of ubiquitin, ubiquitin‐activating enzymes, ubiquitin‐conjugating enzymes, ubiquitin ligases, de‐ubiquitin enzymes, and proteasomes [195]. The effects of these specific components on cells are involved in the pathogenesis of cancer; therefore, they can be used as targets for cancer treatment. The treatment of cancer can be specifically divided into two aspects: treatment for the ubiquitination system and treatment for the proteasome system [196].

In the ubiquitination system, E1, E2, and E3 ligases play important roles in tumor cells. Therefore, the clinical development of these inhibitors has become the focus of myeloma treatment [197]. E1 inhibitors are potentially useful therapeutic agents for patients resistant to proteasome inhibitors as demonstrated by clinical studies. However, the development of E2 inhibitors remains at an exploratory stage. Notable breakthroughs have been made in the development of E3 inhibitors in clinical practice.

E3 inhibitors are widely used in clinical practice. Lenalidomide, thalidomide, and pomalidomide are immunomodulatory agents. Their anti‐tumor effects are mainly achieved through immunomodulatory, anti‐inflammatory effects, and anti‐angiogenesis effects; inhibition of adhesion molecules; anti‐osteoclastic effects; and anti‐tumor cell proliferation effects. PROTAC is a type of targeted protein degradation technology that exhibits good specificity and high degradability, showing promising prospects in the field of malignant tumor treatment [198]. Molecular gel degraders have good prospects for the treatment of cancer. At present, the most effective drugs in the clinical treatment of cancer are proteasome inhibitors [199].

Recent reports indicate that bortezomib, carfilzomib, ixazomib, oprozomib, and other proteasome inhibitors show excellent clinical efficacy [200]. Combination therapy with proteasome inhibitors, such as bortezomib, lenalidomide, and dexamethasone, has also been approved for the treatment of cancer and has achieved a very significant effect [201]. Additionally, proteasome inhibitors can be combined with IMiDS, conventional chemotherapy, and isatuximab. These combined treatment methods have demonstrated good clinical efficacy for cancer treatment [202]. By studying the various mechanisms of action of deubiquitinating enzymes, we found that deubiquitinating enzymes are promising new targets for the treatment of myeloma and have excellent development prospects [203]. However, further research is needed to confirm their clinical applications. UPS‐targeting systems have experienced remarkable progress in the cancer treatment, and there is room for development in the future.

## Author Contributions

Z.L. conceptualized, wrote—reviewed and edited, and administered the project. J.L. wrote the original draft, visualized the study, and gathered resources. Z.M. resources. J.P. validated the study. C.Y. gathered resources. R.F. supervised, acquired funding, and administered the project. All authors contributed to the article and approved the submitted version.

## Ethics Statement

Since this review solely synthesizes existing literature without involving any new experiments, human/animal subjects, or clinical trials, ethics approval is not applicable. All referenced data are publicly available and properly cited.

## Conflicts of Interest

The authors declare no conflicts of interest.

## Data Availability

Data availability is not applicable to this article as no new data were created or analyzed in this study.
